# Genome-Wide Mapping of Loci Explaining Variance in Scrotal Circumference in Nellore Cattle

**DOI:** 10.1371/journal.pone.0088561

**Published:** 2014-02-18

**Authors:** Yuri T. Utsunomiya, Adriana S. Carmo, Haroldo H. R. Neves, Roberto Carvalheiro, Márcia C. Matos, Ludmilla B. Zavarez, Pier K. R. K. Ito, Ana M. Pérez O'Brien, Johann Sölkner, Laercio R. Porto-Neto, Flávio S. Schenkel, John McEwan, John B. Cole, Marcos V. G. B. da Silva, Curtis P. Van Tassell, Tad S. Sonstegard, José Fernando Garcia

**Affiliations:** 1 Departamento de Medicina Veterinária Preventiva e Reprodução Animal, Faculdade de Ciências Agrárias e Veterinárias, UNESP - Univ Estadual Paulista, Jaboticabal, São Paulo, Brazil; 2 Departamento de Zootecnia, UNESP - Univ Estadual Paulista, Jaboticabal, São Paulo, Brazil; 3 GenSys Consultores Associados, Porto Alegre, Rio Grande do Sul, Brazil; 4 Departamento de Apoio, Saúde e Produção Animal, Faculdade de Medicina Veterinária de Araçatuba, UNESP - Univ Estadual Paulista, Araçatuba, São Paulo, Brazil; 5 Division of Livestock Sciences, Department of Sustainable Agricultural Systems, BOKU - University of Natural Resources and Life Sciences, Vienna, Austria; 6 CSIRO - Commonwealth Scientific and Industrial Research Organization, Food Futures Flagship, Brisbane, Queensland, Australia; 7 Centre for Genetic Improvement of Livestock, University of Guelph, Guelph, Ontario, Canada; 8 Centre for Reproduction and Genomics, AgResearch, Invermay, Mosgiel, Otago, New Zealand; 9 Animal Improvement Programs Laboratory, ARS-USDA - Agricultural Research Service - United States Department of Agriculture, Beltsville, Maryland, United States of America; 10 Bioinformatics and Animal Genomics Laboratory, Embrapa Dairy Cattle, Juiz de Fora, Minas Gerais, Brazil; 11 Bovine Functional Genomics Laboratory, ARS-USDA - Agricultural Research Service - United States Department of Agriculture, Beltsville, Maryland, United States of America; Auburn University, United States of America

## Abstract

The reproductive performance of bulls has a high impact on the beef cattle industry. Scrotal circumference (SC) is the most recorded reproductive trait in beef herds, and is used as a major selection criterion to improve precocity and fertility. The characterization of genomic regions affecting SC can contribute to the identification of diagnostic markers for reproductive performance and uncover molecular mechanisms underlying complex aspects of bovine reproductive biology. In this paper, we report a genome-wide scan for chromosome segments explaining differences in SC, using data of 861 Nellore bulls (*Bos indicus*) genotyped for over 777,000 single nucleotide polymorphisms. Loci that excel from the genome background were identified on chromosomes 4, 6, 7, 10, 14, 18 and 21. The majority of these regions were previously found to be associated with reproductive and body size traits in cattle. The signal on chromosome 14 replicates the pleiotropic quantitative trait locus encompassing *PLAG1* that affects male fertility in cattle and stature in several species. Based on intensive literature mining, *SP4*, *MAGEL2*, *SH3RF2*, *PDE5A* and *SNAI2* are proposed as novel candidate genes for SC, as they affect growth and testicular size in other animal models. These findings contribute to linking reproductive phenotypes to gene functions, and may offer new insights on the molecular biology of male fertility.

## Introduction

Reproductive performance has a high economic value in beef cattle, because fertility affects generation intervals, the intensity of selection pressure that can be applied to the population, and the amount of product that can be sent to the market [1]. Furthermore, reproductive wastage is a major reason for culling beef cows.

Domestic cattle are composed by two interfertile species: the humpless taurine cattle (*Bos taurus*) and the humped indicine or zebu cattle (*Bos indicus*). Indicine breeds, such as Nellore cattle, form the majority of the beef herds in tropical and subtropical countries. Zebus generally take longer to reach puberty than taurines [2], making the improvement of reproductive performance an impending challenge in the production systems of these regions of the world.

Scrotal circumference evaluated at yearling (SC) is the most recorded reproductive trait in breeding programs for beef cattle, as the trait is inexpensive and easy to measure [3], is highly heritable [4], and is associated with testis development, quantitative and qualitative semen parameters [5], age at puberty in bulls and related heifers [6], [7], heifer pregnancy [1], and body weight [8]. Consequently, SC is used in these programs as a major indicator of precocity and fertility.

Characterizing genomic regions that explain differences in SC in *B. indicus* can contribute to the identification of reproductive performance informative molecular markers to assist breeding, as well as to the mapping of loci implicated in reproductive biology. In this paper, we analyzed data of estimated breeding values (EBV) from 861 Nellore bulls genotyped for over 777,000 single nucleotide polymorphism (SNP) markers. We aimed at identifying putative genomic regions explaining differences in SC in *B. indicus* cattle via genome-wide mapping.

## Materials and Methods

### Ethical statement

Local ethical committee approval was not necessary in the present study, because phenotypic data were obtained from a database [9], and DNA samples used for genotyping were obtained from commercialized semen straws.

### Animals and phenotypes

Estimated breeding values for SC were obtained from routine genetic evaluations [9], comprising data from 542,918 animals born between 1985 and 2011, and raised in 243 grazing-based Brazilian herds. Scrotal circumference in yearlings (around 18 months of age) was measured as recommended by the *Society for Theriogenology* [3]. Genetic evaluation of SC was based on two single-trait animal models, both including a fixed effect of contemporary group (defined as animals from the same herd, born in the same year and season, and belonging to the same management group from birth until yearling), and random effects that included direct additive genetic, maternal additive genetic, maternal permanent environmental and residual error effects. In the first model (SC_A_), the fixed effect of age at SC measurement was included as a covariate. In the second model (SC_AW_), covariates accounting for differences due to age and weight at yearling were included. Estimated breeding values were deregressed by the method described by Garrick *et al*. (2009) [10] and treated as pseudo-phenotypes in the genome-wide mapping analysis.

### Genotyping and data filtering

Only sires widely used via artificial insemination whose accuracies (i.e., square root of reliability, calculated based on prediction error variance estimates) for SC_A_ and SC_AW_ were greater than 0.5 were considered for analysis. A total of 861 progeny-tested Nellore bulls were genotyped for 777,962 SNPs with the Illumina® BovineHD Genotyping BeadChip assay, according to the manufacturer's protocol. This dataset builds on the data reported by Utsunomiya et al. (2013) [11]. As a first filtering criterion, only samples with call rate greater than 0.9 and SNPs with GenTrain score greater than or equal to 0.7 were considered for analysis. Mitochondrial DNA and unmapped markers were also excluded.

As males are hemizygous for both sex chromosomes, observation of heterozygous X and Y genotypes are only possible for SNP probes that hybridize against the XY pseudo-autosomal (PAR) region. As the *UMD v3.1* bovine genome assembly [12] does not allow for clear distinction of PAR markers, all heterozygous X- and Y-linked genotypes were considered as genotyping errors and set to missing. Next, SNPs were removed from the dataset if they did not exhibit minor allele frequency greater than or equal to 0.02 or call rate of at least 0.98. These procedures and many others described later were performed using customized functions and the *base* and the *GenABEL* v1.7-6 packages in *R v2.15.0* [13], [14].

### Genome-wide mapping

We adapted the two-steps *Fast Association Score Test-based Analysis* (FASTA) [15] to compute allele substitution effects accounting for relatedness, population structure and heterogeneity of variance in deregressed EBVs (dEBVs). In the first step, the variance-covariance matrix for the pseudo-phenotypes was estimated using an animal model that included random additive genetic and residual effects. In the second step, the estimated variance-covariance matrix was used to compute allele substitution effects for each SNP via generalized least squares. A detailed description of this analysis can be found in **File S1**.

Next, aiming at mapping loci explaining differences in SC, we investigated chromosome windows where the average phenotypic variance explained by SNPs deviated substantially from the genome background. First, the percentage of phenotypic variance explained by each SNP was calculated as: 
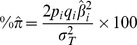



where, relative to SNP 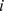, 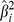 is the estimated allele substitution effect, 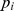 and 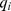 are the allele frequencies, and 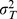 is the total trait variance.

Second, in order to reduce noise and improve mapping, the phenotypic variance explained by SNPs was smoothed across the genome by averaging 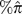 in sliding windows of 1 Mb, sliding 50 kb at a time. Only windows containing at least 10 SNPs were averaged, and we considered as outliers the windows where 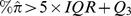, where 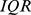 is the interquartile range and 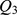 is the third quartile of the distribution. Third, we used *BEDTools v2.12.0* [16] to merge the intervals of overlapping outlier windows. These merged windows were considered as candidate loci for SC.

We assessed the relative mapping resolution gain when using sliding windows instead of single SNPs by calculating the signal-to-noise ratio between the two strategies. For each approach, we let the signal-to-noise coefficient be represented by the reciprocal of the coefficient of variation 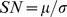, where 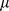 and 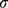 are the mean percentage of phenotypic variance explained and its standard deviation, respectively, by either single SNPs or windows. Then, we calculated the ratio of the signal-to-noise coefficients between strategies as 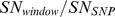.

### Assessment of functional relevance

The *cattle QTLdb* database [17] was examined to find out if any genomic region identified here overlapped with a previously described bovine quantitative trait locus (QTL), in particular those related to body size and reproductive traits. Gene coordinates in the *UMD v3.1* assembly [12] were obtained from the *Ensembl genes 73* database using the *BioMart tool* [18], and overlaps between the boundaries of candidate loci and gene coordinates were determined using *BEDTools v2.12.0* [15]. Finally, we conducted intensive literature review to propose functionally sound candidate genes associated with SC.

## Results

A total of 525,961 SNPs (67.6%) and 861 individuals (100.0%) passed the filtering criteria and were retained in the dataset. After filtering, the average and median gap size between consecutive markers were 5.1 kb and 3.3 kb, respectively, indicating high density coverage of the genome. In spite of the effort and interest to analyze both sex chromosomes, Y SNPs did not present sufficient variability to allow for estimation of allele substitution effects.

**Figure 1:**
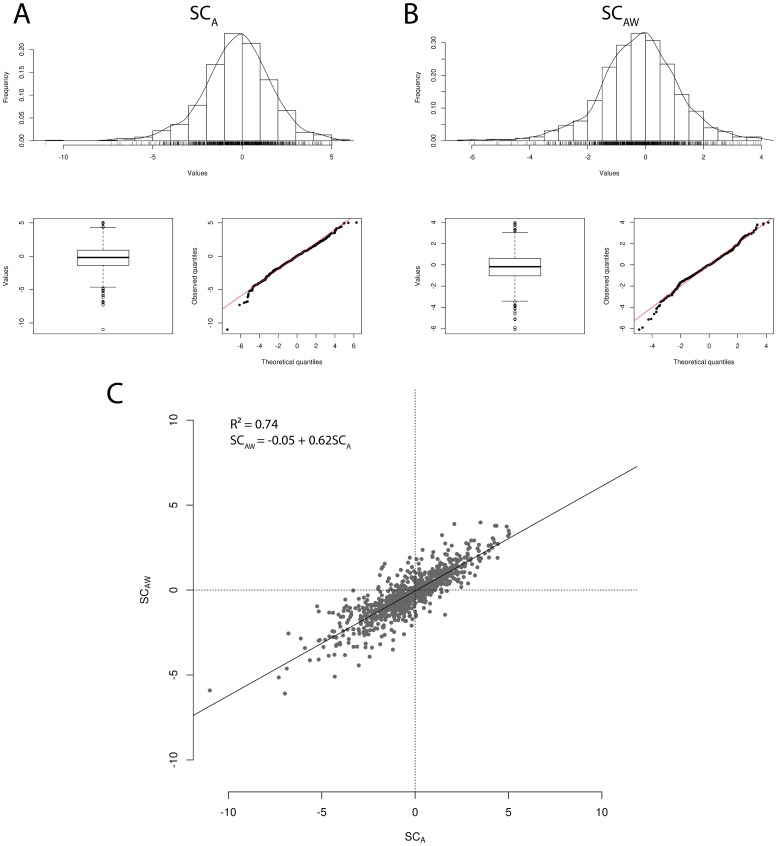
Descriptive statistics for scrotal circumference dEBVs of 861 Nellore bulls. Histograms (top), boxplot (bottom left) and normal quantile-quantile plots (bottom right) are provided for scrotal circumference A) corrected for age (SC_A_) and B) corrected for age and weight at yearling (SC_AW_). A scatter plot illustrating the linear relationship between the two dEBVs is also provided (C).

For both SC_A_ and SC_Aw_, the distributions of pseudo-phenotypes were approximately normal, and the dEBVs for these two traits were fairly correlated, with R^2^ = 0.74 (**Figure 1**). Accuracies were virtually equal for SC_A_ and SC_Aw_ (identical up to the second decimal place), with mean, minimum, median and maximum of 0.84±0.11, 0.51, 0.86 and 0.98, respectively.

**Figure 2:**
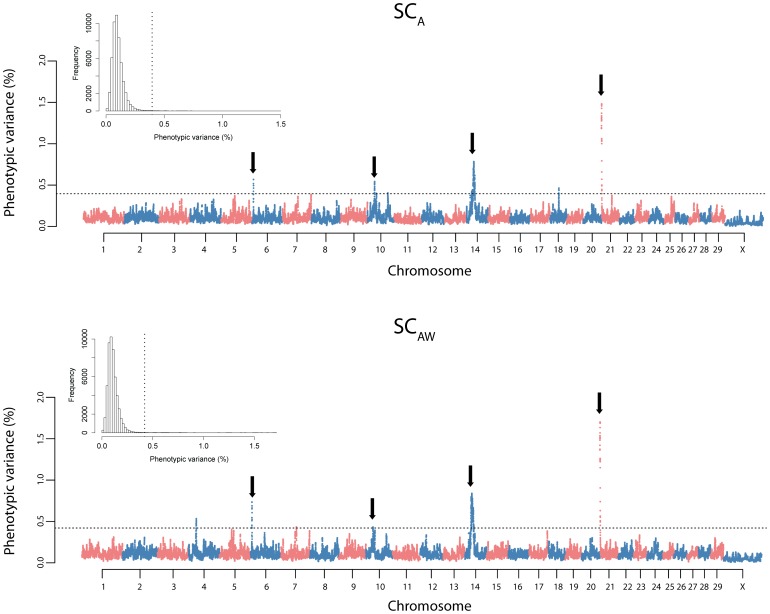
Manhattan plots of scrotal circumference variance explained by SNP windows in Nellore cattle. Pseudo-phenotypes were based on dEBVs corrected for age (SC_A_) and corrected for age and weight at yearling (SC_AW_). Each dot represents a 1 Mb SNP window. Horizontal dashed lines represent adopted thresholds (SC_A_ = 0.40% and SC_Aw_ = 0.42%). Arrows indicate signals shared between the two models. Histograms represent the distribution of phenotypic variance explained by SNP windows, and the dotted vertical line marks the adopted thresholds.

A total of 52,493 SNP windows of 1 Mb were built across the genome, with an average density of 198±59 SNPs per window. The ratio between the signal-to-noise coefficients of sliding windows and single SNPs was 2.54, indicating that the smoothing strategy allowed for a 2.54 fold improvement in mapping resolution (**Figures 2**, **S1** **and** **S2**). Considering the 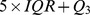 threshold for percentage of phenotypic variance explained (SC_A_ = 0.40% and SC_Aw_ = 0.42%), 236 (0.45%) and 279 (0.53%) windows were declared outliers for SC_A_ and SC_AW_, respectively. After merging overlapping outlier windows, we obtained a total of 8 and 6 candidate loci explaining approximately 4% of the variance in SC_A_ and SC_AW_, respectively (**Table 1**).

**Table 1:** Detected major loci explaining variance in scrotal circumference in Nellore cattle.

Scrotal circumference model	Chromosome	Position start (Mb)	Position end (Mb)	Peak position (Mb)	Segment length (Mb)	Number of SNPs	Average MAF^a^	Average 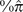 b	Functional candidate gene
Corrected for age (SC_A_)	6	5.20	6.65	5.80–6.00	1.45	42	0.28	0.52	*PDE5A (ENSBTAG00000024888)*
	10	26.90	28.80	28.00	1.90	387	0.23	0.49	*C15ORF55* (ENSBTAG00000014948)
	10	34.80	35.80	35.30	1.00	140	0.19	0.40	*FSIP1 (ENSBTAG00000012015)*
	10	78.85	79.85	79.35	1.00	122	0.20	0.41	-
	14	20.25	21.45	20.90	1.20	310	0.24	0.41	*SNAI2* (ENSBTAG00000013227)
	14	23.40	33.85	29.10	10.45	2236	0.23	0.55	*PLAG1* (ENSBTAG00000004022)
	18	34.55	35.80	35.20	1.25	206	0.22	0.44	*CES4A* (ENSBTAG00000038325)
	21	0.00	2.50	1.20	2.50	127	0.17	1.07	*MAGEL2* (ENSBTAG00000045998)
Corrected for age and weight at yearling (SC_AW_)	4	28.95	30.80	30.15	1.85	301	0.20	0.48	*SP4* (ENSBTAG00000014389)
	6	5.05	6.75	5.80–6.00	1.70	84	0.27	0.63	*PDE5A (ENSBTAG00000024888)*
	7	59.15	60.25	59.75	1.10	158	0.16	0.43	*SH3RF2* (ENSBTAG00000006762)
	10	27.15	28.25	27.70	1.10	209	0.21	0.43	*C15ORF55* (ENSBTAG00000014948)
	14	23.25	35.50	25.00 and 27.00	12.25	2687	0.23	0.63	*PLAG1* (ENSBTAG00000004022)
	21	0.00	2.50	1.20	2.50	127	0.17	1.23	*MAGEL2* (ENSBTAG00000045998)
									

a MAF =  Minor allele frequency.

b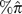 =  Average percentage of phenotypic variance explained by overlapping 1 Mb SNP windows.

**Figure 3:**
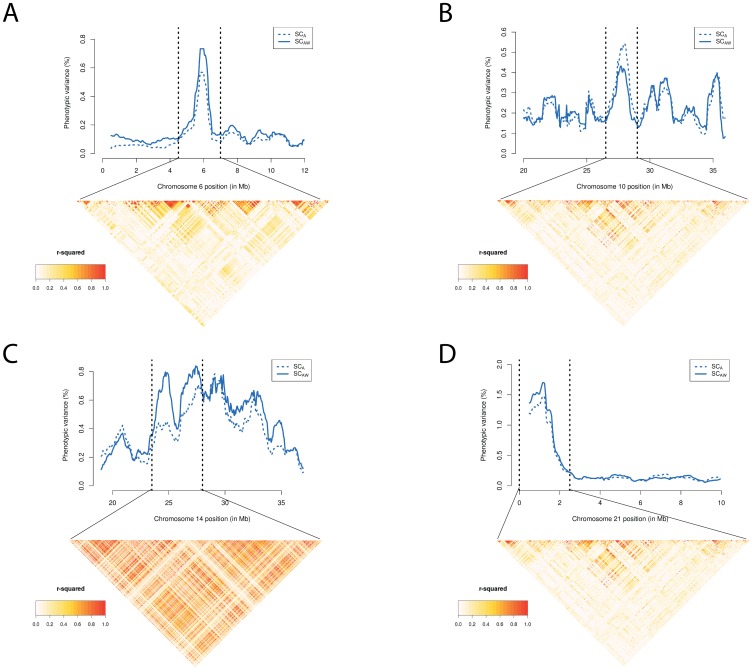
Regional plots of scrotal circumference variance explained by SNP windows in Nellore cattle. Pseudo-phenotypes were based on dEBVs corrected for age (SC_A_) and corrected for age and weight at yearling (SC_AW_). Clear common signals between SC_A_ and SC_Aw_ were found on chromosomes A) 6, B) 10, C) 14 and D) 21. Vertical black dashed lines delimit the regions where the highest variance explained were found. Linkage disequilibrium structure for these regions (bottom) is portrayed as a heatmap of r^2^ values between SNPs.

Overall, results from the genome-wide mapping analysis were strikingly similar between SC_A_ and SC_AW_, indicating that fitting the covariate for weight at yearling in the model did not cause substantial mapping differences. Indeed, four loci were shared between the two traits in chromosomes 6, 10, 14 and 21, respectively (**Figure 3**), which exhibited clear signals in the genome-wide plot of smoothed phenotypic variance explained by SNP windows (**Figure 2**). From the total of six differentially detected loci, one was located nearby the shared locus on chromosome 14 (**Table 1**), and the three SC_Aw_ private loci on chromosomes 10 and 7 were close to be declared outliers for SC_A_ (**Figure 2**).

A total of 285 and 190 genes were mapped against the major loci found for SC_A_ and SC_AW_, respectively, and a total of 309 unique genes were observed (**File S2**). From these, 246 protein coding, 25 snoRNA, 12 snRNA, 9 miRNA, 9 rRNA, 1 misc_RNA and 7 pseudo genes were observed (**File S2**). From this gene list, we filtered 9 functional candidates implicated in growth, testicular size and fertility (**Table 1**), which included: pleiomorphic adenoma gene 1 (*PLAG1*, ENSBTAG00000004022), carboxylesterase 4A (*CES4A*, ENSBTAG00000038325), Sp4 transcription factor (*SP4*, ENSBTAG00000014389), melanoma antigen family L 2 (*MAGEL2*, ENSBTAG00000045998), phosphodiesterase 5A (*PDE5A*, ENSBTAG00000024888), snail family zinc finger 2 (*SNAI2*, ENSBTAG00000013227), nuclear protein in testis (*C15ORF55*, ENSBTAG00000014948), fibrous sheath-interacting protein 1 (*FSIP1*, ENSBTAG00000012015), and SH3 domain containing ring finger 2 (*SH3RF2*, ENSBTAG00000006762).

A total of 76 production and reproduction QTLs, mined from 24 distinct publications, were mapped against the loci found here (**File S3**). The largest trait contingency observed was composed by traits related to body size (43 QTLs), followed by reproductive traits (23 QTLs). Furthermore, the locus detected on chromosome 4 (**Table 1**) mapped against one previously described QTL for SC in Angus cattle (*B. taurus*) [19].

## Discussion

The genome-wide mapping analysis detected positional candidate loci explaining approximately 4% of the dEBVs for SC (**Table 1**). Although this represents only a fraction of the trait variance, this percentage is substantial considering that 180 loci associated with human adult height, a highly heritable and classic polygenic trait, explain only 10% of the phenotypic variance together [20]. This is evidence that multiple loci across the genome are involved in the complex inheritance of SC, and the functional candidate genes filtered here may only scratch the surface of the molecular mechanisms underlying the trait. The dissection of the pathways regulating precocity in *B. indicus* cattle will require multiple studies across breeds and trait models, with intensive multidisciplinary reasoning. Nevertheless, the loci reported here excel from the genome background, and represent important data in the context of bovine reproductive biology.

The region explaining the largest proportion of SC variance in the present study mapped to the beginning of chromosome 21, peaking around 1.5 Mb. The closest gene found in this region was *MAGEL2*. The orthologous human and murine genes regulate normal circadian output, and are highly expressed in the suprachiasmatic nucleus of the hypothalamus [21]. The human *MAGEL2* has been implicated in Prader-Willi Syndrome, a genetic disorder characterized by short stature, low muscle tone, cognitive disabilities, increased food intake, obesity, low levels of insulin and insulin-like growth factor 1 (IGF1), incomplete sexual development, hypogonadism, and male infertility [21], [22]. The disorder manifests when a segment on human chromosome 15, which encompasses seven maternally imprinted genes including *MAGEL2*, presents a deletion or loss of expression of the paternal alleles. Inactivation of the mouse *MAGEL2* alone was shown to lead to abnormalities suggestive of hypothalamic dysfunction similar to the Prader-Willi Syndrome [22].

Of note, variation of copy number gain spanning the interval between 1.57 Mb and 2.99 Mb on bovine chromosome 21 has been found by the comparison of individual whole genome sequence data of Nellore with the *B. taurus* breeds Angus, Holstein and Hereford [23]. As the Prader-Willi Syndrome is caused by loss of the paternal copy of the orthologous sequence in humans, and *MAGEL2* is essential for proper hypothalamic control of growth and fertility [21], association of copy number variants with growth and reproductive traits seems to be a sensible hypothesis to be tested on this chromosome segment.

The locus detected on chromosome 7 encompasses *SH3RF2*. Rubin *et al*. (2010) [24] discovered a deletion removing all but the first exon of the orthologous chicken gene that is associated with body weight, and demonstrated that strong selection caused the deletion to reach fixation in a high growth lineage. Interestingly, using a mouse model of Prader-Willi syndrome, Stefan *et al*. (2005) [25] found that loss of expression of the *MAGEL2* region induces upregulation of *SH3RF2* and its flanking genes *TCERG1*, *LARS*, *RBM27* and *GPR151*. As both the *MAGEL2* and the *SH3RF2* regions were flagged in the present study, a trans-acting regulatory mechanism involving the loci on chromosomes 7 and 21 found here is likely to underlie SC variation. Hence, these signals are plausible candidates for weight and male fertility traits in Nellore cattle.

We identified a candidate locus on chromosome 14 with the highest percentage of phenotypic variance explained mapping to positions 25 Mb and 27 Mb. Fortes *et al*. (2013) [26] reported associations for IGF1 at 6 months and SC at 12 months in young Brahman bulls (*B. indicus*) in an overlapping region around 25 Mb, which was previously shown to correlate with age of Brahman bulls when they achieve 26 cm of SC [27]. This region has been well characterized in taurine cattle as harboring several human orthologues affecting stature and growth [28], especially *PLAG1* [29]. The locus has also been found to be associated with birth weight in Nellore cattle, and suggested to shelter polymorphisms with pleiotropic effects on traits that correlate with body size [11]. Furthermore, some first evidences for pleiotropism in body size and fertility traits in the *PLAG1* region have been recently found in Brahman cattle [30].

Although the human stature orthologues flanking 25 Mb are appealing candidates for SC, the chromosome 14 signal found here comprises a large segment spanning from 20.25 Mb to 35.85 Mb. This may be evidence that multiple genes and variants within this region are involved. For instance, *SNAI2* is located around 21.58 Mb and encodes for Slug (also known as Slugh), a Zinc-finger transcription factor that when mutated in mice produces males with testicular atrophy and marked decrease in seminiferous tubules sizes [31]. Although these mice are able to copulate, their offspring are small. Also, Fortes *et al*. (2013) [26] showed that ability to produce sperm at 18 months in Brahman bulls is not as significant around 25 Mb, and exhibits signals of association shifted towards the 35 Mb position instead.

Another possible justification for a signal coming from such a large chromosome segment is a long range linkage disequilibrium persistency within the region. In fact, we found a strong linkage disequilibrium structure underpinning the signal (**Figure 3**), which may be hampering the localization of the true locus involved. In either case, these evidences together support the entire chromosome segment identified here as a key region affecting growth and fertility traits in cattle.

The locus detected on chromosome 6 is located 124 kb downstream of *PDE5A*. The phosphodiesterase encoded by *PDE5A* is substantially expressed in the testis, and mice overexposed to inhibitors of this protein present testicular tissue alterations, including decreased testis weight, degeneration, and atrophy of the seminiferous epithelium [32]. This genomic region also shelters genes that interact with other proteins previously linked to small testis size. For instance, the protein encoded by *MAD2* belongs to the mitotic checkpoint complex, and is recruited by the mitotic kinase Bub1. A residue change in the catalytic loop of Bub1 was shown to lead to male subfertility, with marked reduction in testicular size [33].

Several QTLs mapping to the loci detected here were related either to body size or reproductive traits that are associated with SC. In particular, the peak on chromosome 4 mapped against one previously reported QTL for SC [19], which encompasses *SP4*. This gene encodes for a zinc finger transcription factor that is predominantly expressed in the brain, but is also detectable in the testicular tissue [34], [35]. Göllner *et al*. (2001) [36] showed that *SP4*-knockout mice develop until birth without obvious abnormalities, but two-thirds of them die within 4 weeks after birth and the remaining one-third present growth retardation. Surviving male mice exhibit reduced testis size, although complete spermatogenesis can be observed. Surviving female mice exhibit small-sized thymus, spleen and uterus, and all mice show pronounced delay in sexual maturation. As *SP4*-knockout mice present growth retardation mainly after birth, it is likely that variations in the bovine *SP4* affect body size and testicular growth from birth to yearling age, but they are unlikely to affect fetal development or spermatogenesis. Moreover, the evidence of delayed sexual maturation and reduced testicular size in surviving *SP4*-knockout mice is consistent with the known positive correlation between SC and precocity in cattle.

The functional candidate gene surrounding the peak on chromosome 18 was *CES4A*, a hydrolase member of the carboxylesterase large family (enzyme class EC 3.1.1.1.-), also known as *CES6*, *CES8* or Hydrolase A. Carboxylesterases act in the transesterification of a broad spectrum of substrates, and play an important role in the metabolism of endogenous lipid and foreign compounds such as drugs and pesticides [37]. Hydrolase A is known to be expressed in several tissues, including the testis [38]. Esterase activity (EC 3.1.1.-) has been found to be abundant in the testis and associated with androgen production [39]. Two intronic SNPs in the human *CES4A* were found to be correlated with high density lipoprotein levels (HDL) in an association analysis deposited in the *dbGaP* database (www.ncbi.nlm.nih.gov/gap, accession: pha002900.1, accessed on 21 Oct 2013), conducted in an expanded population sample from the original 1966 Northern Finland Birth Cohort (NFBC66) study [40]. Also, testosterone treatment of aging men with hypogonadism was demonstrated to lower HDL levels, and the mechanism underlying this relationship and its role in coronary disease risk have been targets of debate and controversy [41]. These evidences together point to *CES4A* as a functional candidate gene affecting differences in SC in Nellore cattle, and the underlying mechanism may involve the dynamics between HDL and androgen levels.

Some of the genes lying within the loci detected nearby positions 28 Mb and 35 Mb on chromosome 10 are related to the testicular tissue, but their association with scrotal circumference is unclear. The nuclear protein in testis gene (*C15ORF55* or *NUTM1*) is mainly known by its involvement with midline organs carcinoma [42]. The fibrous sheath-interacting protein 1 (*FSIP1*) was shown to bind to Akap4 during spermatogenesis in order to form the fibrous sheath of the sperm flagellum [43]. The peak found around 79.35 Mb on chromosome 10 did not reveal an appealing functional candidate gene, but the region overlaps QTLs for dystocia, fetal death and birth weight (**File S3**). Further investigation of these loci is needed to elucidate if they contribute to phenotypic variation in SC, as well as to clarify the molecular mechanisms underlying this contribution.

## Conclusions

In summary, this is believed to be the first study applying a high-density SNP panel in a genome-wide survey of loci affecting scrotal circumference in Nellore cattle, which contributes with important preliminary data to the dissection of molecular mechanisms regulating precocity in the bovine species. The loci identified here harbor known and novel functional candidate genes affecting scrotal circumference in *B. indicus* cattle. Fine mapping of these signals with whole genome sequence data and hypothesis-driven experiments may shed light on the genes and networks underlying phenotypic variation in fertility traits in cattle. In a broader perspective, as the majority of the genes found in eutherian mammals are orthologous, further investigation of these loci in cattle may offer new insights on complex aspects of mammalian reproductive biology.

## Supporting Information

Figure S1 **Manhattan plot of SC_A_ variance explained by single SNPs in Nellore cattle.**
(TIF)

Figure S2 **Manhattan plot of SC_AW_ variance explained by single SNPs in Nellore cattle.**
(TIF)

File S1 **Supporting methods: extended methods for weighted FASTA.**
(PDF)

File S2 **Genes mapping against the loci detected for scrotal circumference in Nellore cattle.**
(XLS)

File S3 **QTLdb hits for scrotal circumference corrected for age (SC_A_) and age and weight (SC_AW_).**
(XLS)
