# Supplementary figures and images for: Genome-Wide Mapping of Loci Explaining Variance in Scrotal Circumference in Nellore Cattle
Source: PLoS One. 2014 Feb 18;9(2):e88561. doi: 10.1371/journal.pone.0088561 (PMC3928245; doi:10.1371/journal.pone.0088561)

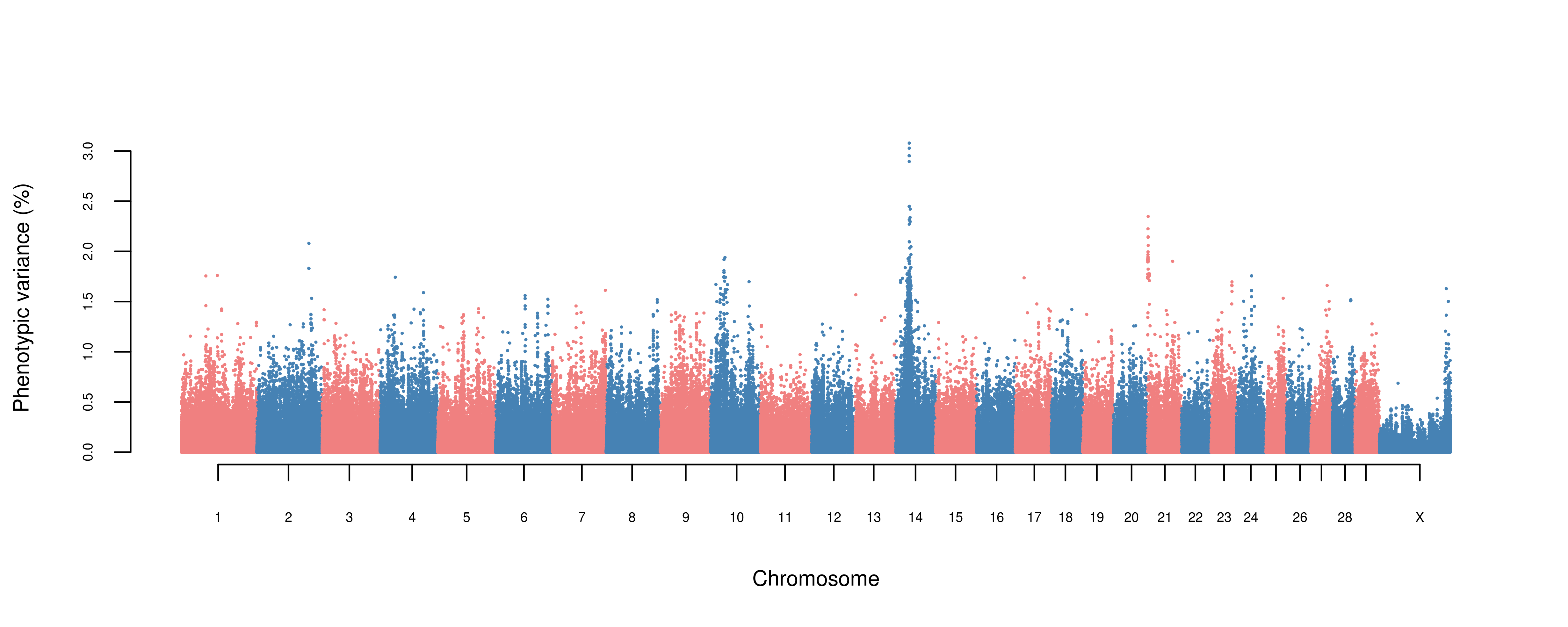

Supplement: Figure S1 — Manhattan plot of SCA variance explained by single SNPs in Nellore cattle. (TIF) [file pone.0088561.s001.tif]

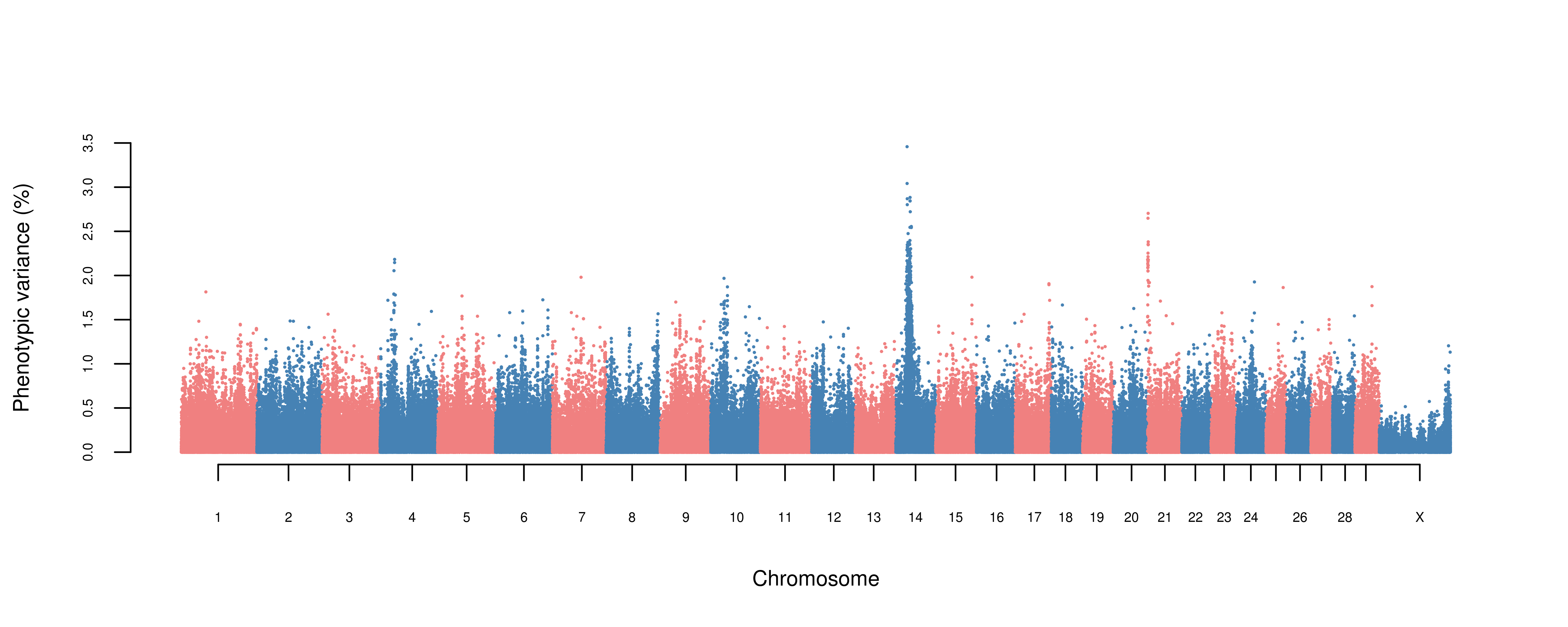

Supplement: Figure S2 — Manhattan plot of SCAW variance explained by single SNPs in Nellore cattle. (TIF) [file pone.0088561.s002.tif]
