# Supplementary material for: Genome-Wide Mapping of Loci Explaining Variance in Scrotal Circumference in Nellore Cattle
Source: PLoS One. 2014 Feb 18;9(2):e88561. doi: 10.1371/journal.pone.0088561 (PMC3928245; doi:10.1371/journal.pone.0088561)
Supplement: File S1 — Supporting methods: extended methods for weighted FASTA. (PDF) [file pone.0088561.s003.pdf]

Extended methods for weighted FASTA

The standard FASTA

Variance-components models are the gold standard for genome-wide association analysis of single nucleotide polymorphism (SNP) markers accounting for relatedness and population substructure. However, fitting all model parameters for every tested SNP makes the process computationally demanding. In order to overcome this problem, approximation approaches have been proposed, which divide the estimation of parameters into two steps: first, a variance-components model is fitted to the data; then, the significance of each marker is either obtained from score tests corrected for the variance-covariance matrix (Chen & Abecasis, 2007; Zhang *et al*., 2010; Kang *et al*., 2010; Lippert *et al*., 2011) or least squares regressions using residuals as the dependent variable (Aulchenko *et al*., 2007; Amin *et al*., 2007).

The *Fast Association Score Test-based Analysis* (FASTA) method (Chen & Abecasis, 2007) comprises fitting a variance-components model to the data in order to obtain the variance-covariance matrix for the phenotypes, which is then used to compute allele substitution effects for each tested SNP. The variance-components model is based on the polygenic model:

$$y = X\beta + u + \varepsilon \quad \textbf{[1]}$$

where y is the vector of phenotypes observed for n individuals, X is a nxk design matrix of k covariates, β is a column vector of size k of fixed effects of covariates, and u and ε are vectors of unobserved random additive genetic and residual effects, respectively.

The polygenic model involves partitioning the total trait variance σ_T^2 in two components: variance due to genetic differences among individuals, namely additive genetic variance σ_u^2, and a residual variance σ_ε^2. Additive genetic effects are assumed to follow a $MVN(0, \Phi\sigma_u^2)$, where Φ is a relationship matrix. The model specifies that the random residual effect for each individual is normally distributed with mean zero and variance σ_ε^2. These residuals are assumed to be independent between individuals,

and the joint distribution of residuals is defined as $MVN(0, I\sigma_{\varepsilon}^{2})$, where I is an identity matrix. In this setting, variances are assumed to be equal among individual phenotypes, and the variance-covariance matrix is defined as:

$$\Omega = \Phi\sigma_{u}^{2} + I\sigma_{\varepsilon}^{2} \quad \textbf{[2]}$$

The log-likelihood function of the model is then specified as:

$$log(L) = -\frac{n}{2} log(2\pi) - \frac{1}{2} log|\Omega| - \frac{1}{2}(y - X\beta)^{T}\Omega^{-1}(y - X\beta) \quad \textbf{[3]}$$

Maximum likelihood estimates for each model parameter are obtained from this function by using an optimization algorithm. Next, the estimated σ_{u}^{2} and σ_{ε}^{2} are used in **[3]** to calculate the variance-covariance matrix at the point of maximum likelihood. Then, for each SNP, the allele substitution effect and its variance are obtained using generalized least squares equations:

$$\hat{\beta}_{SNP} = (\tilde{g}'\Omega^{-1}\tilde{g})^{-1}\tilde{g}'\Omega^{-1}\tilde{y} \quad \textbf{[4]}$$

$$var(\hat{\beta}_{SNP}) = (\tilde{g}'\Omega^{-1}\tilde{g})^{-1} \quad \textbf{[5]}$$

Where $\hat{\beta}_{SNP}$ and $var(\hat{\beta}_{SNP})$ are the estimated allele substitution effect and its variance, respectively; $\tilde{g} = g - E(g)$, where g is the vector of observed genotypes for a given marker coded as 0, 1 or 2 reference alleles, and $E(g)$ is a genotype mean; and $\tilde{y}$ is the vector of dependent phenotype residuals $\tilde{y} = y - X\hat{\beta}$ (phenotype adjusted for the estimated fixed effects).

Adapting FASTA to account for heterogeneity of variance in dEBVs

Following Garrick *et al*. (2009), a deregressed estimated breeding value (dEBV) represents a pseudo-phenotype that summarizes all the information available on the individual and its relatives, as if it was a single observation. In this special case, variances are unequal among individual pseudo-phenotypes as the number of repeated measures or observations of relatives for each individual varies. Thus, it is necessary to adapt the polygenic model to allow for heterogeneity of variance among individuals. This can be achieved by replacing the diagonal of the identity matrix I in

the residual variance matrix by individual weights that are proportional to the estimate errors. Now, residual random effects are assumed to follow a $MVN(0, R\sigma_\varepsilon^2)$, where R is a diagonal weight matrix.

The main objective in maximizing the likelihood in **[3]** is to obtain estimates for the variance components and random/fixed effects of the model. However, it is easy to show that the polygenic model in FASTA is equivalent to the animal model:

$$y = X\beta + Zu + \varepsilon \quad \textbf{[6]}$$

where Z is a design matrix of order nxn that relates phenotypes to random animal effects. The random effects are assumed to be distributed as:

$$\begin{bmatrix} u \\ \varepsilon \end{bmatrix} \sim MVN\left(\begin{bmatrix} 0 \\ 0 \end{bmatrix}, \begin{bmatrix} \Phi\sigma_u^2 & 0 \\ 0 & R\sigma_\varepsilon^2 \end{bmatrix}\right) \quad \textbf{[7]}$$

This model can be fitted using the mixed model equations (MME) developed by Henderson (1973):

$$\begin{bmatrix} X'R^{-1}X & X'R^{-1}Z \\ Z'R^{-1}X & Z'R^{-1}Z + \Phi^{-1}\lambda \end{bmatrix} \begin{bmatrix} \hat{\beta} \\ \hat{u} \end{bmatrix} = \begin{bmatrix} X'R^{-1}y \\ Z'R^{-1}y \end{bmatrix} \quad \textbf{[8]}$$

Where λ is the variance ratio $\sigma_\varepsilon^2/\sigma_u^2$. Now, variance components can be estimated via restricted (residual) maximum likelihood (REML), and $\hat{\beta}$ and $\hat{u}$ can be simultaneously estimated. The variance-covariance matrix for the phenotypes is then defined as $V = Z'(\Phi\sigma_u^2)Z + R\sigma_\varepsilon^2$. As dEBVs are single observations, matrix Z is an identity matrix, so the model in **[6]** can be rewritten as $y = X\beta + u + \varepsilon$, and the variance-covariance matrix becomes

$$V = \Phi\sigma_u^2 + R\sigma_\varepsilon^2 \quad \textbf{[9]}$$

Note that $R = I$ when variances are equal among individual phenotypes, in which case $V = \Omega = \Phi\sigma_u^2 + I\sigma_\varepsilon^2$, and the animal model is equivalent to the original polygenic model used in FASTA. Thus, we used REML to estimate variance components and MME to estimate fixed and random effects in the first step of FASTA, allowing for fitting

phenotypes with unequal variances. The allele substitution effect and the variance of each SNP were then obtained as in the original FASTA approach, following **[4]** and **[5]**.

Obtaining a relationship matrix from SNP data

The diagonal elements of matrix Φ are individual variances, which can be expressed as $1 + F$, where F is the inbreeding coefficient (Astle & Balding, 2009). The off-diagonal elements of this matrix are covariances between individuals, which can be expressed as twice their kinship coefficient. Methods for obtaining unbiased positive semi-definite estimates of the kinship matrix from SNP data have been proposed by Amin *et al*. (2007), VanRaden (2008) and Astle and Balding (2009). In human genetics, the most widely used estimator for the kinship coefficient is the one described by Amin *et al*. (2007), which is defined as follows:

$$\hat{f}_{i,j} = \frac{1}{L}\sum_{l=1}^{L}\frac{(g_{l,i}-p_l)(g_{l,j}-p_l)}{p_l(1-p_l)} \quad \textbf{[10]}$$

where $\hat{f}_{i,j}$ is the estimated genomic kinship between individuals i and j, L is the total number of loci used for the calculation, p_l is the major allele frequency for locus l, and $g_{l,i}$ and $g_{l,j}$ are the locus l genotypes for individuals i and j, respectively (coded as 0, 1/2 and 1, for minor allele homozygote, heterozygote and major allele homozygote, respectively). Although in a different notation, this is the same estimator as proposed by Astle and Balding (2009):

$$\hat{K} = \frac{1}{L}\sum_{l=1}^{L}\frac{(g_l-2p_l1_n)(g_l-2p_l1_n)'}{4p_l(1-p_l)} \quad \textbf{[11]}$$

where $\hat{K}$ is the estimated kinship matrix, L is the total number of loci, g_l is the vector of genotypes for locus l (coded as 0, 1 or 2 reference alleles), and p_l is the reference allele frequency for locus l. If we let $z_l = g_l - 2p_l1_n$, and $k_l = 4p_l(1-p_l)$, formula **[11]** can be rewritten as:

$$\hat{K} = \frac{1}{L}\sum_{l=1}^{L}\frac{z_l z_l'}{k_l} \quad \textbf{[12]}$$

The livestock genetics and animal breeding communities are more familiar with the estimator proposed by VanRaden (2008):

$$\hat{G} = \frac{ZZ'}{k} \textbf{ [13]}$$

where $\hat{G}$ is the estimated kinship matrix, k is a scaling parameter defined as $2\sum_{l=1}^{L} p_l(1-p_l)$, p_l is the reference allele frequency for locus l, and Z is a centered genotype matrix. This matrix is obtained by subtracting from the genotype matrix M (coded as -1, 0 and 1 for reference allele homozygote, heterozygote and reference allele homozygote, respectively) the matrix P, whose elements of column l are equal to $2(p_l - 0.5)$.

Recognize that estimators **[12]** and **[13]** are closely related, but **[13]** scales the kinship matrix across all loci, while **[12]** scales markers individually. Figures **S1.1** and **S1.2** illustrate the estimates obtained for the Nellore dataset by the three methods. The correlation between the kinship coefficients obtained from the method described by VanRaden (2008) and either Amin *et al*. (2007) or Astle and Balding (2009) was 0.975. As the three methods yielded similar estimates, we decided to use the estimator proposed by Amin *et al*. (2007), as implemented in the GenABEL package v1.7-6.

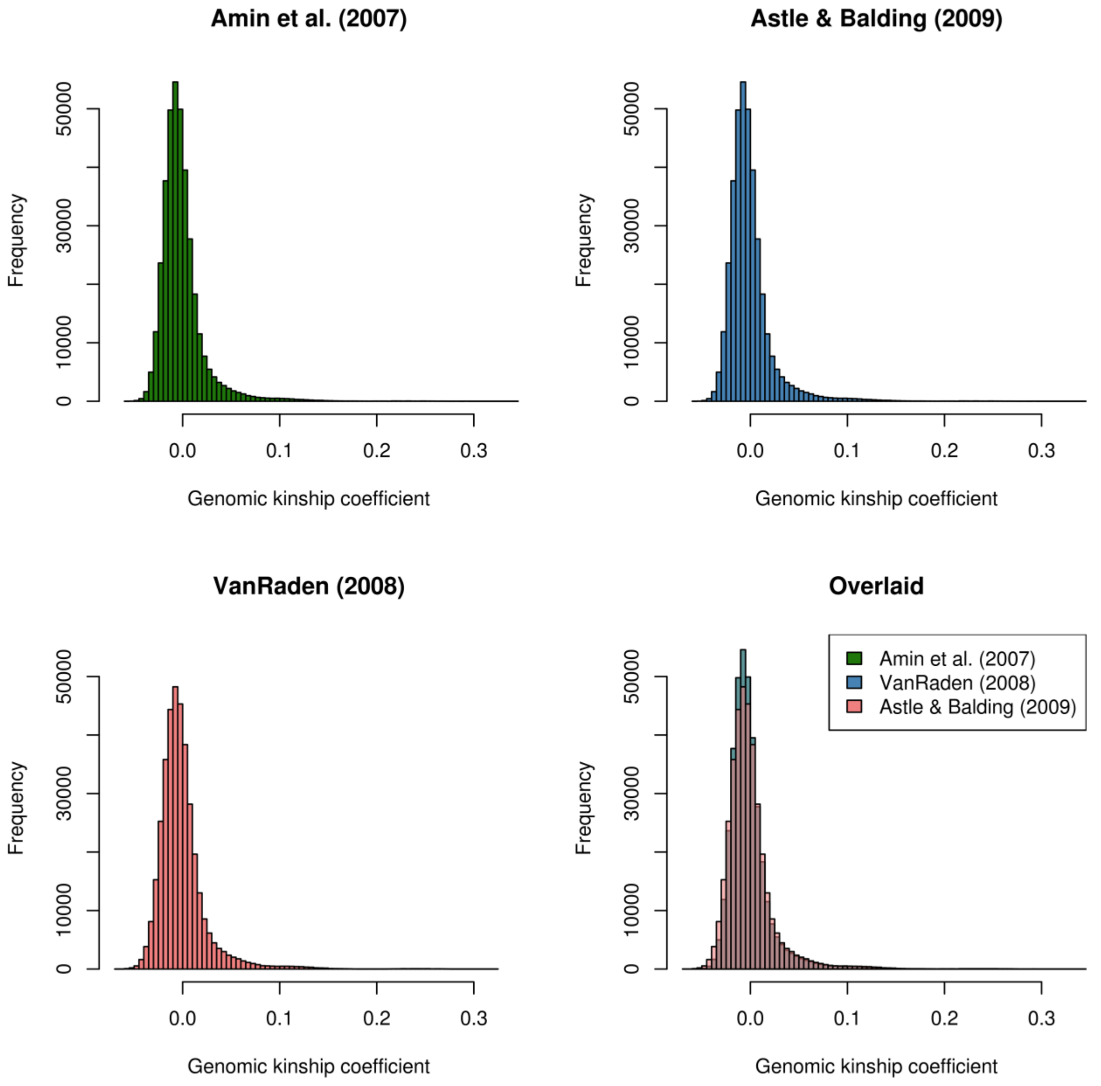

Figure S1.1. Histograms of genomic kinship coefficients estimated for the Nellore dataset. Estimates were obtained based on 10,000 randomly sampled autosomal SNPs.

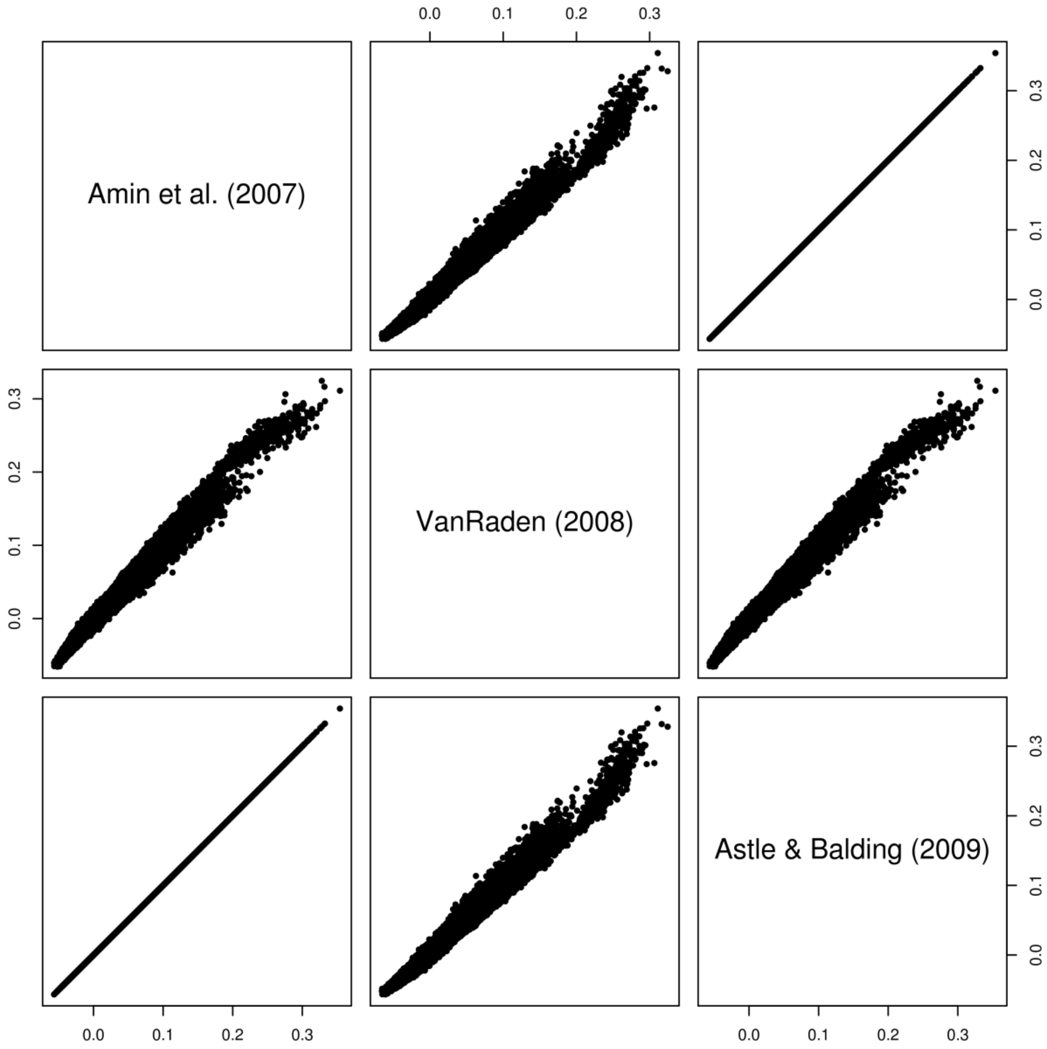

Figure S1.2. Scatterplots of genomic kinship coefficients estimated for the Nellore dataset. Estimates were obtained based on 10,000 randomly sampled autosomal SNPs

Choosing appropriate weights for dEBVs

The choice of weights to be used can vary according to the nature of the response variable being analyzed. In the case of dEBVs, Garrick *et al*. (2009) proposed the following weights to account for heterogeneity of variance:

$$w_i = \frac{(1-h^2)}{\left[\left(c+\frac{1-r_i^2}{r_i^2}\right)h^2\right]} \quad \textbf{[14]}$$

where c is the assumed proportion of the genetic variance not explained by markers due to partial genome coverage and incomplete linkage disequilibrium between markers and causal variants, h^2 is the estimated heritability of EBVs before deregression, and r_i^2 is the reliability of the dEBV of animal i. These weights are diagonal elements of the inverse of the scaled residual variance matrix $R\sigma_\varepsilon^2$, with σ_ε^2 being factored out before inversion. Hence, the weight matrix R can be expressed in the non-inverted form as:

$$R = \begin{bmatrix} 1/w_1 & \dots & 0 \\ \vdots & \ddots & \vdots \\ 0 & \dots & 1/w_i \end{bmatrix} \quad \textbf{[15]}$$

Garrick *et al*. (2009) argued that the choice of a value for c can be made by assessing a range of values or by estimating c from validation analyses. In practice, they showed that the impact of the assumed value of c is to influence the relative value of individuals with accurate information, in comparison to individuals with less reliable information. When $c = 0$, weighting by w is equivalent to weighting observations by the inverse of their variances, which in the case of dEBVs can be approximated by $1/(1 - r^2)$. As the use of too large a value of c would result in little contrast between dEBVs with low and high accuracy, and the use of too small a value of c would result in excessive emphasis on dEBVs with high accuracy, we decided to fix c at 0.5.

References

1. Amin N, Van Duijn CM, Aulchenko YS. A genomic background based method for association analysis in related individuals. *PLoS One* 2007; 2:e1274.

2. Astle W, Balding DJ. Population structure and cryptic relatedness in genetic association studies. *Statistical Science* 2009; **24**:451-71.

3. Aulchenko YS, de Koning D-J, Haley C. Genomewide rapid association using mixed model and regression: a fast and simple method for genomewide pedigree-based quantitative trait loci association analysis. *Genetics* 2007; 177:577-85.

4. Chen WM, Abecasis GR. Family-based association tests for genomewide association scans. *American Journal of Human Genetics* 2007; 81:913-26.

5. Garrick DJ, Taylor JF, Fernando RL. Deregressing estimated breeding values and weighting information for genomic regression analyses. *Genetics Selection Evolution* 2009; 41:55.

6. Henderson CR. Sire evaluation and genetic trends. *Journal of Animal Science* 1973; 10-41.

7. Kang HM, Sul JH, Service SK, Zaitlen NA, Kong SY, Freimer NB *et al*. Variance component model to account for sample structure in genome-wide association studies. *Nature Genetics* 2010; 42:348-54.

8. Lippert C, Listgarten J, Liu Y, Kadie CM, Davidson RI, Heckerman D. FaST linear mixed models for genome-wide association studies. *Nature Methods* 2011; 8:833-5.

9. VanRaden PM. Efficient methods to Compute Genomic predictions. Journal of Dairy Science 2008; 91:4414-23.

10. Zhang Z, Ersoz E, Lai CQ, Todhunter RJ, Tiwari HK, Gore MA *et al*. Mixed linear model approach adapted for genome-wide association studies. *Nature Genetics* 2010; 42:355-360.
